# Modelling asynchrony in phenology considering a dynamic representation of meteorological variables

**DOI:** 10.7717/peerj.18653

**Published:** 2025-02-11

**Authors:** Rubén de la Torre Cerro, Gourav Misra, Emily Gleeson, Guy Serbin, Jesko Zimmermann, Fiona Cawkwell, Astrid Wingler, Paul Holloway

**Affiliations:** 1 Department of Geography, University College Cork, Cork, Ireland; 2 Environmental Research Institute, University College Cork, Cork, Ireland; 3 School of Natural and Environmental Sciences, Newcastle University, Newcastle upon Tyne, United Kingdom; 4 National Centre for Geocomputation, Maynooth University, Kildare, Ireland; 5 Department of Computer Science, Maynooth University, Kildare, Ireland; 6 Research and Applications Division, Met Éireann, Dublin, Ireland; 7 EOanalytics Limited, Dublin, Ireland; 8 Department of Agrifood Business and Spatial Analysis, Rural Economy and Economic Development Programme, Teagasc Ashtown Research Centre, Dublin, Ireland; 9 School of Biological, Earth and Environmental Sciences, University College Cork, Cork, Ireland

**Keywords:** Phenological mismatch, Ecological network, Co-existence, Interaction, Asynchrony

## Abstract

Shifts in the timing of phenological events across many taxa and ecosystems are a result of climate change. Within a trophic network, phenological mismatches between interlinked species can have negative impacts for biodiversity, ecosystems, and the trophic network. Here we developed interaction indices that quantify the level of synchrony and asynchrony among groups of species in three interlinked trophic levels, as well as accounting for a dynamic representation of meteorology. Insect first flight, vegetation green-up and arrival of migrant birds were the phenological indicators, obtained from a combination of spatially and temporally explicit species observations from citizen science programmes and remote sensing platforms (*i.e.*, Landsat). To determine phenological shifts in interlinked taxa we created and applied several phenological indices of synchrony-asynchrony, combining information from the phenological events and critical time windows of meteorological variables. To demonstrate our method of incorporating a meteorological component in our new interaction index, we implemented the relative sliding time window analysis, a stepwise regression model, to identify critical time windows preceding the phenological events on a yearly basis. The new indices of phenological change identified several asynchronies within trophic levels, allowing exploration of potential interactions based on synchrony among interlinked species. Our novel index of synchrony-asynchrony including a meteorological dimension could be highly informative and should open new pathways for studying synchrony among species and interaction networks.

## Introduction

Phenology is the study of cyclic and seasonal phenomena in organisms, such as leaf unfolding, leaf senescence, flowering of plants, animal migration, and timing of the breeding season. Quantifying temporal changes in phenology is imperative for biodiversity conservation, as phenological variations can result in changes in ecosystem functioning and services (Peñuelas & Filella, 2001; Peñuelas, Rutishauser & Filella, 2009; Le Quéré et al., 2018). The impact of climate change on phenology is widely studied (Gordo & Sanz, 2010; Jones & Cresswell, 2010; Primack et al., 2009; Roberts et al., 2015; Gordo & Sanz, 2005; Visser, Holleman & Gienapp, 2006; O’Neill et al., 2012; Gordo & Sanz, 2006; Dunn & Møller, 2014; Bateman et al., 2016; Chmura et al., 2023), with results typically demonstrating the relationship between phenology and the variation in climatic drivers. The effect of temperature rise is widely linked to advanced phenological events (Van De Pol et al., 2016; Irons et al., 2017), and given projected global increases in temperature (Seneviratne et al., 2021), there is the potential for significant phenological change across ecosystems.

Phenological delays for a single species can have an extended impact across the trophic network due to the complexity of interlinked ecosystems. Although phenological advances resulting from climate change are well documented (*i.e.,* earlier flowering and leaf unfolding in spring—Mayor et al., 2017; Primack et al., 2009; Rafferty & Ives, 2011), sensitivity and response to changes in meteorological conditions are expected to vary within groups and species, as well as in space and time. Therefore, phenological asynchronies between different trophic levels are expected (*e.g.*, flowering time and pollinator activity; bird migration and insect development) given species specific phenological sensitivity to weather and climate (Thackeray et al., 2016; Kharouba et al., 2018; Youngflesh et al., 2023). Phenological asynchronies result in a reduction in pollination services and supress interactions among species (Both et al., 2009); however, to-date methods for studying phenological interactions alongside climate drivers are scarce (de la Torre Cerro & Holloway, 2021).

Studies investigating spatiotemporal asynchronies in phenological events have predominantly focused on monitoring changes of a single species, or an array of species, that are usually linked through the trophic network (Gordo & Sanz, 2006; Jones & Cresswell, 2010; O’Neill et al., 2012; Reed et al., 2013; Dunn & Møller, 2014). Spatial asynchronies in the phenology of these interactor species can provide important information on distributional ranges, while temporal asynchronies provide insight into potential trophic cascades. Trophic indices of asynchrony have been traditionally developed between producers and primary consumers or two directly connected levels using either direct (biotic interactions) or indirect (co-existence) methods (Post & Forchammer, 2008; Mayor et al., 2017). However, the exploration of trophic synchrony-asynchrony across more than two levels through an index is rare (de la Torre Cerro & Holloway, 2021).

The variation of phenological events is often statistically related to a choice of environmental variables (*i.e.,* climate); however, these models might not capture the actual variation simply due to an incorrect selection of abiotic drivers or their spatiotemporal scale (Van De Pol et al., 2016). Selecting the appropriate temporal scale of environmental variables determining phenological sensitivity is neither an easy task, nor should it be a factor of minor concern (Van De Pol & Bailey, 2019). Moreover, phenological sensitivity to the environment varies across species, meaning interspecific interactions can be altered due to asynchronies between trophic levels. This can result in negative effects on individual species’ fitness, which could translate into implications at community and ecosystem level (Donoso et al., 2016; Timberlake, Vaughan & Memmott, 2019). Therefore, selecting the most informative environmental variables with an appropriate temporal resolution, time window, and spatial extent is vital to enhance model performance and predictions of a particular event of study (Van De Pol et al., 2016; Holloway, Kudenko & Bell, 2018; Simmonds, Cole & Sheldon, 2019). Given the overarching effect of these environmental drivers on phenological events and subsequent interactions, any interaction index must consider the role of the environment within their quantification.

While interaction indices have been widely used (Donoso et al., 2016; Oleques, Overbeck & de Avia Jr, 2017; Powell et al., 2024), there persists a need to investigate phenological synchrony-asynchrony across multiple trophic levels. Moreover, given the importance of environmental variables in determining the timing of these events, research is needed to identify whether such variables can be incorporated into interaction indices to better inform our understanding of phenology and support biodiversity conservation. Here we address this research gap by using time series records of phenological events to model species synchrony-asynchrony through the trophic network, while simultaneously including a high-resolution meteorological dimension. This novel interaction index, which accounts for (a) multiple trophic levels and (b) environmental variables, should open new pathways for studying synchrony-asynchrony among species and interaction networks under climate change. Secondary to this methodological development, our research also permits the opportunity to ask questions related to meteorological drivers and their critical time windows associated with phenological events over a 11-year period and whether these windows of interaction alter or change across the three trophic levels.

## Methods

### Study area and species

Ireland is situated in the north-west of Europe in the North-East Atlantic basin, presenting a temperate maritime climate (McCarthy, Gleeson & Walsh, 2015). Future annual temperatures (mean, maximum, minimum) and rainfall (annual, seasonal) are expected to increase in both the short-term (2040) and long-term (2100) (O’Brien & Nolan, 2023; O’Brien et al., 2024; Wang et al., 2024), which will have implications for the timing of phenological events. The landscape is fragmented with a patchy configuration of grassland pastures, agricultural areas, and forest, with the predominant types of woodland being broad-leaved, bog, coniferous, mixed, and transitional woodland-shrub (Carlier et al., 2021).

Changes in phenology in the Republic of Ireland (hereafter Ireland) are well documented, particularly advanced spring phenology for different species groups (O’Neill et al., 2012; Donnelly, Salamin & Jones, 2006; Donnelly, Yu & Liu, 2015). For example, earlier time of emergence and extended flight season of moths (O’Neill et al., 2012), advancements and/or delays in time of arrival and departure of migrant birds as well as extended length of stay (Donnelly, Geyer & Yu, 2015) and earlier start and duration of growing season in trees (Donnelly, Salamin & Jones, 2006). Consequently, we have selected Ireland as a case study to apply our models for phenological research while emphasizing these methodologies have a broad scope and can be applied to phenological systems elsewhere.

We explored phenological synchrony/asynchrony for three different trophic levels: woodland vegetation, insects (lepidoptera; butterflies and moths), and birds. Species selection for insects and birds was performed to select common and migrant species, respectively, while screening for species with a high number of observations and preferably those that are interlinked through the trophic network. Table 1 shows the common names and codes used for animal species and woodland vegetation habitats. Indicator species for the woodland habitats (canopy, shrub, and field layer) are listed by Perrin et al. (2008).

**Table 1:** List of species and woodland vegetation habitats shown by group, illustrating the common name of each species and the code each species or vegetation group was given in our study. **GVW** (*Pieris napi*), **LW** (*Pieris brassicae*), **MB** (*Maniola jurtina*), **PC** (*Aglais io*), **RA** (*Vanessa atalanta*), **RI** (*Aphantopus hyperantus*), **SMW** (*Pieris rapae*), **ST** (*Aglais urticae*), **SPW** (*Pararge aegeria*), **BM** (*Opisthograptis luteolata*), **CMC** (*Dysstroma truncate*), **DA** (*Apamea monoglypha*), **ET** (*Selenia dentaria*), **FS** (*Ochropleura plecta*), **HD** (*Agrotis exclamationis*), **LY** (*Noctua pronuba*), **SS** (*Diarsia rubi*), **WE** (*Spilosoma lubricipeda*), **BS** (*Hirundo rustica*), **GW** (*Sylvia communis*), **NW** (*Oenanthe oenanthe*) **SW** (*Acrocephalus schoenobaenus*) and **WW** (*Phylloscopus trochilus*).

Species/Group		Code	Species/Group		Code
Vegetation Group			Butterfly Species		
		Green Veined White	GVW
Bog Woodland	BW	Large White	LW
Mixed Broadleaf/Conifer Woodland	MBC	Meadow Brown	MB
(Mixed) Broadleaf Woodland	MBW	Peacock	PC
Oak-Ash-Hazel Woodland	OAH	Red Admiral	RA
Oak-Birch-Holly Woodland	OBH	Ringlet	RI
Wet Pedunculate Oak-Ash Woodland	WOA	Small Tortoiseshell	ST
Wet Willow-Alder-Ash Woodland	WAA	Small White	SMW
		Speckled Wood	SPW
Migrant Birds			Moth Species	Brimstone Moth	BM
				Common Marbled Carpet	CMC
	Barn Swallow	BS		Dark Arches	DA
	Greater Whitethroat	GW		Early Thorn	ET
	Northern Wheatear	NW		Flame Shoulder	FS
	Sedge Warbler	SW		Heart & Dart	HD
	Willow Warbler	WW		Large Yellow	LY
				Small Square	SS
				White Ermine	WE

### Species data

Start of season (hereafter, “green-up”, “GU”) dates were calculated from national scale Landsat data. These data were obtained in a pre-processed form from the United States Geological Survey (USGS) EROS Science Processing Architecture On Demand Interface (ESPA) (https://espa.cr.usgs.gov/). Spatially and temporally explicit data on insects (first flight) and birds (date of arrival) were collated from citizen science projects. Butterfly data were obtained from The Irish Butterfly Monitoring Scheme from the National Biodiversity Data Centre (https://www.biodiversityireland.ie), and moth data from MothsIreland (http://www.mothsireland.com), and bird data from eBird (2017) (Sullivan et al., 2009) and BirdTrack (https://www.bto.org/our-science/projects/birdtrack).

### Meteorological data

Three meteorological drivers were selected for inclusion as explanatory variables in the statistical models: maximum temperature (TMAX), minimum temperature (TMIN), and total precipitation (TPPT). These variables were calculated using hourly values obtained from Met Éireann’s ReAnalysis (MÉRA, Whelan, Gleeson & Hanley, 2018; Gleeson, Whelan & Hanley, 2017) data set. This high resolution regional reanalysis spans an area covering Ireland, the UK and part of northern France on a 2.5 km horizontal grid from 1981 to August 2019. Daily precipitation totals and maximum temperatures were computed using the sum and maximum value respectively between the period 9 UTC to 9 UTC the following day. Daily minimum temperatures were computed using the minimum temperature for the same period, but the value is assigned to the following day. We utilised a high-resolution (30 m) digital elevation model (DEM) dataset to apply a correction to the temperatures (daily TMAX was too low and daily TMIN was too high on average), to account for a mismatch between the orography in the HARMONIE-AROME (Bengtsson et al., 2017) model used for MÉRA and the actual orography (Gleeson & Whelan, 2020). The DEM dataset was firstly projected onto the MÉRA grid using the nearest neighbour method in Climate Data Operators (CDO). The height differences between the MÉRA orography and those of the DEM were used along with the international standard atmosphere lapse rate of 6.49 K/km, as defined by the International Civil Aviation Organization (ICAO), to apply temperature corrections based on the height differences between the model and DEM orographies.

### Data processing

Green-up dates in the form of Julian day were extracted from daily Normalised Difference Vegetation Index (NDVI) values derived from Landsat imagery using the half-amplitude method (Misra et al., 2018; White et al., 2009). Pre-processed Landsat time series NDVI data masked for cloud cover for the years 2007–2018 (See Zenodo for detailed methodological steps). The National Survey of Native Woodlands 2003–2008 (Perrin et al., 2008) and Ancient Long-Established Woodlands (Perrin & Daly, 2010) survey-based polygons were intersected to maximise the information available within each dataset obtained from the National Parks and Wildlife Service, Ireland (NPWS, 2012; NPWS, 2019). The resulting intersected polygons consisted of areas that have been pristine for the last two centuries, have 100% coverage of trees and information on the dominant species. These polygons were used to extract mean values of NDVI per raster layer and aggregated to monthly maximum values to reduce the frequency of missing observations due to the previous pre-processing (Fensholt & Proud, 2012). Residual missing observations in the NDVI time series of each polygon were then filled using mean annual values, also referred to as filling with climatology (Kandasamy et al., 2013). Each NDVI time series was subsequently smoothed to remove spikes in the time series data and interpolated to daily values using a LOESS function (Hufkens et al., 2019; Yuan et al., 2021).

First flight (FF) and date of arrival (DA) were calculated for insects and birds, respectively. To ensure sufficient species records to allow a robust sample size for use in the statistical models and to overcome uncertainty associated with whether the first observation of species sightings from the citizen science datasets was reflective of observer effort, we re-scaled all data to a spatial resolution of 10 km × 10 km. We required a minimum of 3 species sightings per 10 km grid to be included in the statistical analysis, with a minimum of 20 grids required per species and per year (see Zenodo). The number of grids represents the sample size, herein referred to as N. This resulted in us merging the eBird and BirdTrack datasets to ensure sufficient data of bird arrival. We took the first (*i.e.,* earliest) date of the species sighting as the date of the phenological event. We should note that the date in which each phenological event is registered for the first time in a grid might differ within grids and within years, and, consequently, the meteorological conditions in which the event took place might be quite different to those experienced by the same species taking place in another grid.

### Synchrony-asynchrony index

To evaluate phenological asynchrony/synchrony between different trophic levels we used the following interaction indices:

(1) \begin{eqnarray*}A{L}_{(a,b)}=(D{A}_{ai}-F{F}_{bj})\end{eqnarray*}

(2) \begin{eqnarray*}A{L}_{(a,c)}=(D{A}_{ai}-G{U}_{ck})\end{eqnarray*}

(3) \begin{eqnarray*}A{L}_{(b,c)}=(F{F}_{bj}-G{U}_{ck})\end{eqnarray*}

where a, b, and c refer to the trophic levels incorporated in the analysis, respectively, which are level 3 or secondary consumers (*i.e.,* birds), level 2 or primary consumers (*i.e.,* insects) and level 1 or producers (*i.e.,* woodland vegetation). *DA* refers to the date of arrival of birds, *FF* refers to the date of first flight of insects, and *GU* refers to green up of vegetation. *i*, *j*, and *k* refer to the different species incorporated in the index for trophic levels *a*, *b*, and *c*, respectively.

When the phenological event for the higher trophic level takes place after the lower levels, this index results in a positive number. This infers that the higher trophic level is either exhibiting synchrony or delayed synchrony with the lower levels. Conversely, when it takes place before, this index results in a negative number. This infers that the higher trophic level is exhibiting asynchrony, or in other words birds are arriving before the insects take flight.

For ease of interpretation, *AL* values were standardised between a consistent maximum and minimum, with the maximum and minimum values for each group of species within a trophic level. Below is an example for trophic levels a and b.

(4) \begin{eqnarray*}A{L}_{(a,b)}^{{^{\prime}}}= \frac{ \left( A{L}_{(a,b)}-min \left( A{L}_{(a,b)} \right) \right) }{(\max \nolimits \left( A{L}_{(a,b)} \right) -\min \nolimits \left( A{L}_{(a,b)} \right) )} \end{eqnarray*}

Values close to 0 reflect asynchrony, 0.5 reflect perfect synchrony, and 1 reflect delayed synchrony. Delayed synchrony means that species are undertaking their phenological events in the anticipated order, but the delay between them may still result in trophic cascades.

To assess interaction across the multiple trophic levels, we integrated Eqs. (1) and (3) to estimate asynchrony among three trophic network levels using a qualitative integrated (a)synchrony index (QAI) Eq. (5):

(5) \begin{eqnarray*}QAI=A{L}_{(a,b)}^{{^{\prime}}}{|}A{L}_{(b,c)}^{{^{\prime}}}\end{eqnarray*}

We took the median value for AL’_(a,b)_ and AL’_(b,c)_ and substituted the numeric values by “A” (asynchrony) when AL’ was lower than 0.5 or “S” (synchrony) when AL’ was equal or greater than 0.5. We then put together those qualitative categories as shown in Eq. (5) with the aim to easily identify whether synchrony was preserved over trophic levels, by comparing bird-insect — insect-vegetation. We carried out a total of ninety combinations for the QAI through 3 trophic levels. Qualitative combinations of QAI were: AS, AA, SS or SA. For example, SS was reported when green up occurred before first flight, which subsequently occurred before date of arrival, while AS was reported where green-up occurred before first flight, but first flight occurred after date of arrival.

### Interaction index of climate window movement

To evaluate changes in the sensitivity to meteorological variables and synchrony-asynchrony, we developed the Interaction Index of Climate Window Movement (IICWM). This novel approach to quantifying synchrony-asynchrony includes a meteorological dimension building on our AL index. For the purposes of this index, the use of climate and meteorology can be considered synonymous, as the approach could be extended from daily resolution weather data to decadal climate data. The meteorological dimension is the critical time window for which a particular meteorological event resulted in the ‘best’ statistical model of the phenological event calculated using a relative sliding time window (SWR) analysis from Climwin (Bailey & Van De Pol, 2016), in R (R Core Team, 2014). Climwin fits all potential time windows within a selected range for each phenological record and employs nested for-loops varying the start and end time of these windows. In our case we wanted to test both the effect of spring phenology, but also if our models could capture autumn signals. Therefore, we chose a temporal range of between 0 and 180 days prior to the event. The meteorological dataset is then subset, only taking values that match the tested window, providing a “window open” value and “window close” value. We ran the relative sliding time window analysis for each species and year, across the study period. For a full methodological account of the sliding time window analysis, please see Zenodo.

We then combined the synchrony-asynchrony index calculated through Eq. (1) with the variation in the date of the opening critical time window for TMIN, TMAX, and TPPT, for each bird-insect interaction.

(6) \begin{eqnarray*}I\text{ICWM}= \left( \frac{A{L}_{(a,b)}}{WO} \right) \end{eqnarray*}

where WO refers to window open, calculated using Eq. (7). We used window open as this parameter represents the initial date in which the model captures a signal from the meteorological cue.

(7) \begin{eqnarray*}WO= \left( WO[TMINa]-WO[TMINb] \right) \nonumber\\\displaystyle  + \left( WO[TMAXa]-WO[TMAXb] \right) +(WO[TPPTa]-WO[TPPTb])\end{eqnarray*}

with the value a combination of the three environmental variables, TMIN, TMAX and TPPT run iteratively for trophic levels a and b. Using the non-standardised AL_(a,b)_ and WO values for each particular year and species combination we identified three possible categorisations (+,-,*) and two scenarios:

• Positive (+) IICWM represents a bird-insect combination that is synchronous, and situations where bird time windows take place further in the past and/or insect windows take place closer to first flight (scenario A).
• Asterisk (*) IICWM represents a bird-insect asynchronous combination together with bird time windows taking place closer to DA or insect time windows taking place further back before first flight (scenario B).
• Negative (-) IICWM will be returned in two cases: (1) when a particular bird-insect combination is asynchronous in combination with scenario A, or (2) when a synchronous combination occurred together with scenario B.

## Results

### Phenological events from 2008–2018

**Figure 1:**
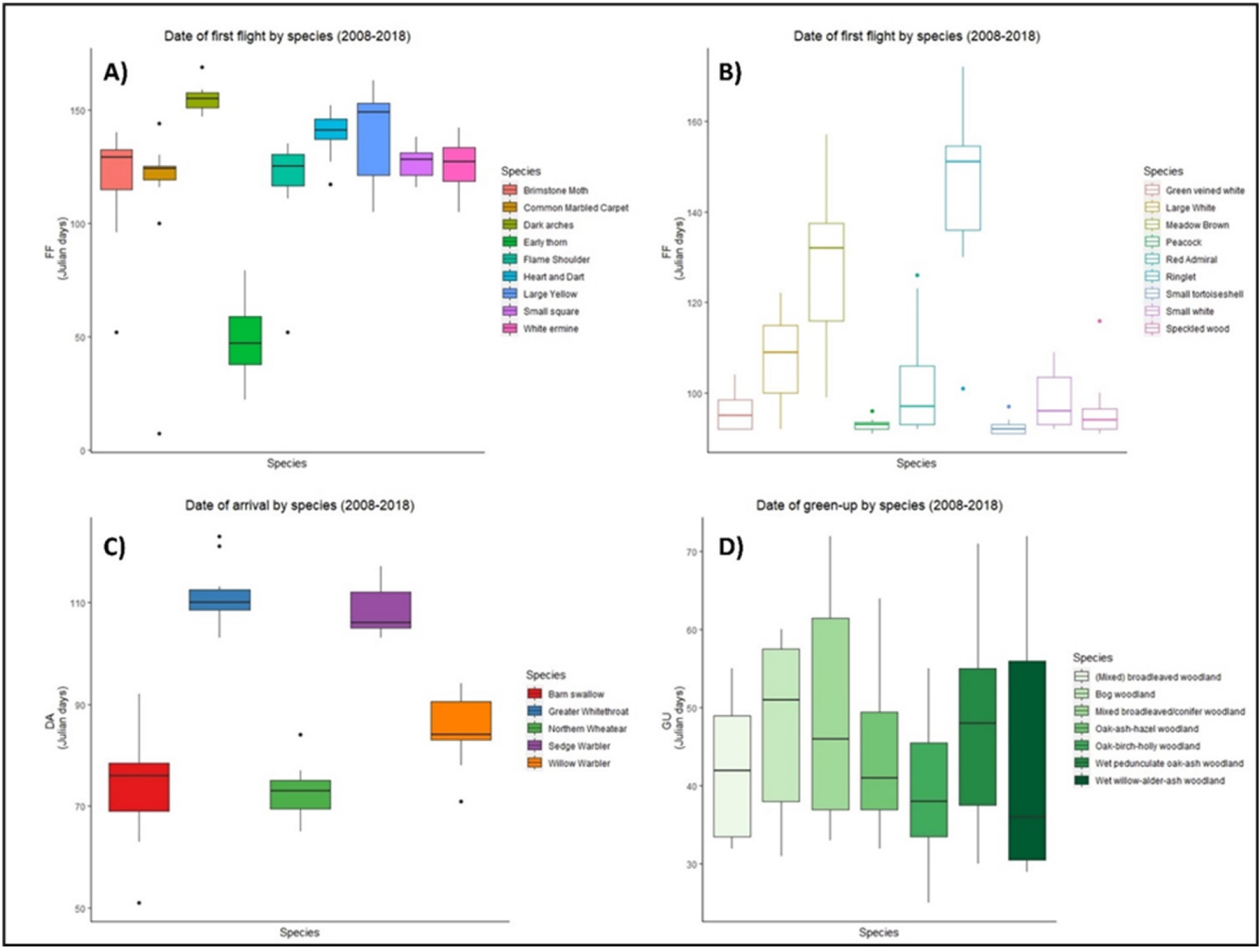
Date of phenological events across the species between 2008–2018. Date of first flight for moths (A) and butterflies (B), date of arrival of migrant birds (C), and green-up dates (D) for the period 2008–2018 at national level in Ireland. X axis represents each species within groups, Y axis represents the date of the year, in ordinal number, in which the events took place. Boxes represent Julian days (ordinal days) and whiskers represent standard deviation, showing median value for each date range (horizontal line inside each box). Different colours represent different species within each group as shown in their respective legends.

There was high variability in the timing of first flight, date of arrival, and green-up between 2008–2018 for all groups of species (Fig. 1). Five out of nine moth species took their first flight between days 120–140 (Fig. 1A), while first flight among butterflies was more variable (Fig. 1B). Four species of butterfly initiated first flight before day 100, while six species showed similar median first flight values (Fig. 1B). In the case of birds, barn swallow, and northern wheatear showed an early spring arrival while greater whitethroat and sedge warbler showed arrivals later in the spring. Three species, willow warbler, sedge warbler, and greater whitethroat presented an early median date of arrival (Fig. 1C). Vegetation green-up dates showed the greatest variation, yet median dates were similar for four out of seven vegetation groups (Fig. 1D).

When phenological events were analysed per species (Zenodo), certain trends emerged, but the consistent result was varying phenology. For example, green-up dates showed great variation during the period 2008–2018, with four vegetation groups (BW, MBC, OAH, and WAA) advancing their phenology from 2008, two groups (MBW and OBH) delaying their phenology, while one group (WOA) remained constant. Five moth species (BM, DA, ET, HD, and WE) had a later first flight in 2018 than in 2008, while three (CMC, FS, and SS) showed an earlier first flight across the decade. A similar result was observed in butterflies, with five species presenting a later first flight in 2018. Arrival of migrant birds also fluctuated, but date of arrival generally advanced over the decade for all species, except for the greater whitethroat.

**Figure 2:**
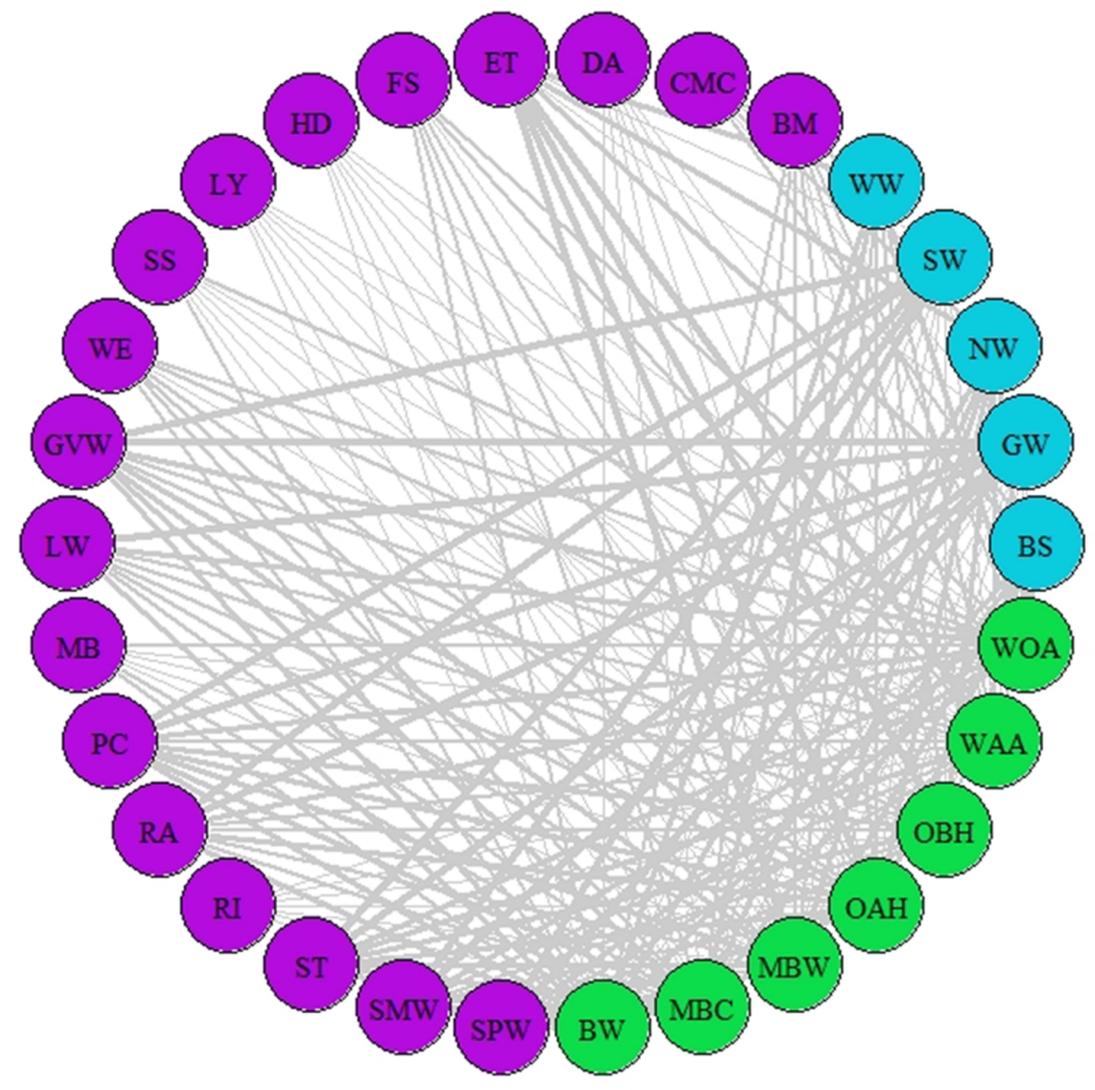
Network of synchrony. Species represented are those that were considered as synchronous by the different AL’ indices (AL’ ranged between 0.5–1). Different colours represent different trophic levels; blue colour represents migrant birds, purple colour represent butterflies and moths, green colour represents vegetation. Lines represent synchrony between pairs of species. Line thickness represents synchrony degree, the thicker the line the more synchronous the relationship (value closer to 0.5, considered as total synchrony by our indices).

**Figure 3:**
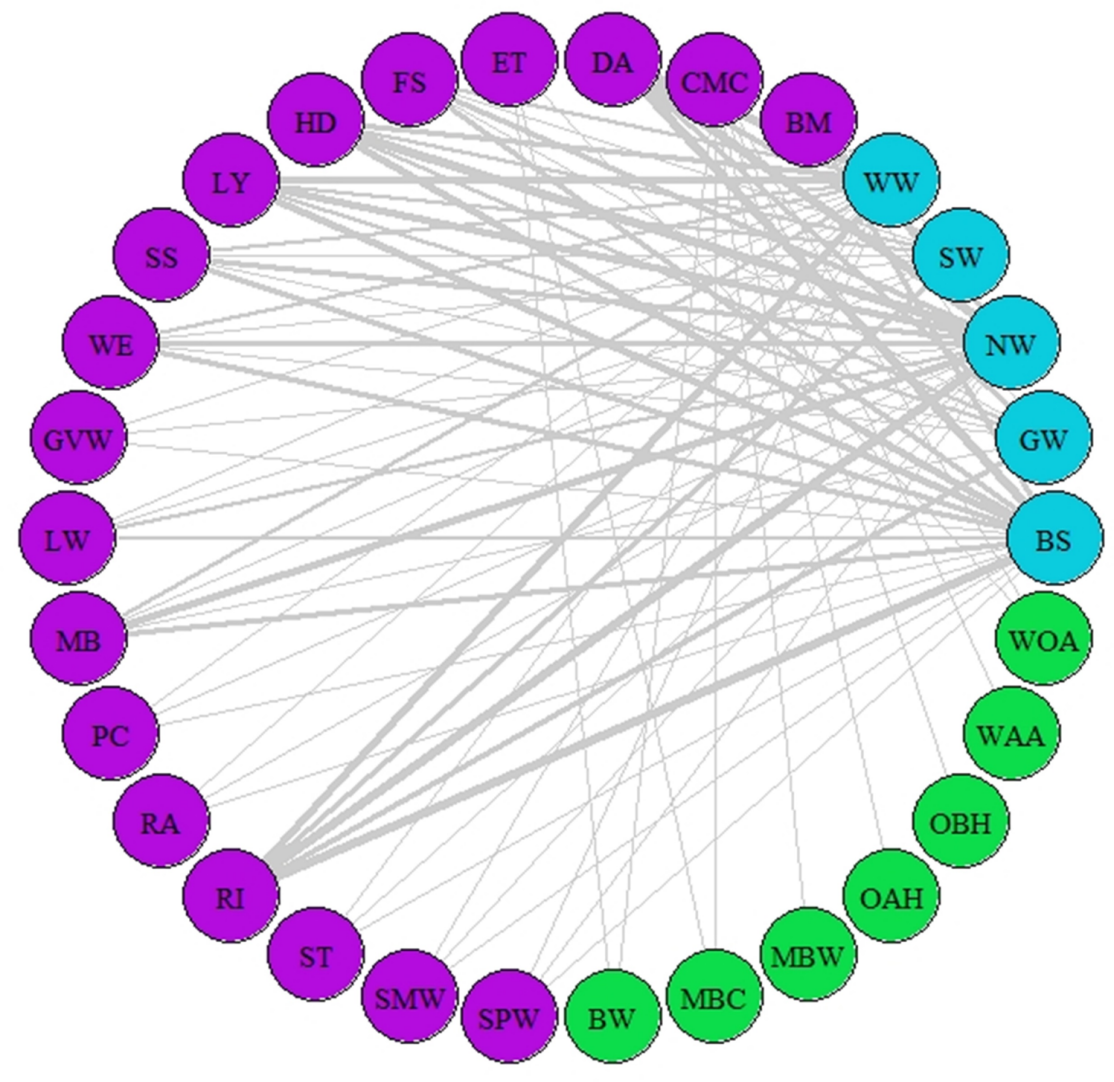
Network of asynchrony. Illustration of asynchrony degree among our study species according to AL’ indices. Different colours represent different trophic levels; blue colour represents migrant birds, purple colour represent butterflies and moths, green colour represents vegetation. Lines represent asynchrony between pairs of species. Line thickness represents asynchrony degree, the thicker the line the more asynchronous the relationship (value closer to 0, considered as total asynchrony by our indices) while narrower lines represent lower degree of asynchrony.

### Synchrony-asynchrony indices of the trophic levels

The indices of synchrony-asynchrony between all birds and insects (AL_(3,2)_), insects and woodland vegetation (AL_(2,1)_), and birds and woodland vegetation (AL_(3,1)_) were performed for 251 species combinations. Median values across 2008–2018 showed that 77 species combinations were asynchronous (AL’ < 0.5) and 174 were synchronous (AL’ > 0.5). The most asynchronous interactions included barn swallow, northern wheatear, sedge warbler, greater whitethroat, willow warbler, common marbled carpet, and early thorn.

A network of potential species interactions at the national level for all 30 species was developed using the median AL’ scores from 2008–2018 (Figs. 2–3). Species that were synchronous (Fig. 2) are potentially more likely to interact, but thinner lines represent delayed synchrony. Species that were asynchronous (Fig. 3) show those that are occurring in the ‘wrong’ order, with thicker lines showing a longer time discrepancy. Birds reported the greatest asynchrony (Fig. 3) with several delayed relationships (*i.e.,* thick lines) reported. For example, barn swallow showed generalised asynchrony with AL’_(3,2)_ ranging between 0.41–0.12 with all but two insect species, common marbled carpet (0.7) and early thorn (0.63). The common marbled carpet showed asynchrony with all vegetation groups with AL’_(2,1)_ ranging from 0.47–0.43. Early thorn was asynchronous with three vegetation groups, bog woodland, mixed broadleaved/conifer woodland, and wet pedunculate oak-ash woodland, with AL’_(2,1)_ ranging between 0.489–0.49.

### Interaction index of climate window movement

#### Meteorological drivers & time windows of phenological events

Our climwin models illustrated a marked interannual variation in the timing of phenological events. The opening and closing of relevant temporal windows were estimated to influence the three meteorological variables at group and species level (Zenodo). For example, in the case of woodland vegetation, all three meteorological variables showed similar estimated influences for all seven types of vegetation, and Pc indicated statistical significance across drivers and windows (Pc < 0.5), as shown in Table 2 (see Zenodo for a definition of Pc). Data for all time windows across all thirty species and their Pc estimates are shown in Zenodo. Model coefficients showed a negative relationship with increased TMAX, TMIN, and TPPT and green-up dates, indicating that an increase of 1 degree in temperature (either maximum or minimum) and one mm^3^ of TPPT over the identified time window is expected to advance green-up between 0.25 and 10 days.

**Table 2:** Climwin results for OAH vegetation group showing yearly time windows in which green-up was most influenced by each one of our meteorological variables. Window open reflects the number of days prior to GU in which a driver started to influence GU, and takes place further back in time, while window close (also shown in days prior GU) always takes place after window open, closer to the phenological event. GU represents the Julian day of the year in which green-up took place at national level (in ordinal date). N means sample size, number of grids in which GU was measured. Asterisks denote statistical significance showed by climwin Pc values.

**Oak-ash-hazel woodland**			**TMIN**		**TMAX**		**TPPT**	
**Year**	**N**	**GU**	**W Open**	**W Close**	**W Open**	**W Close**	**W Open**	**W Close**
2008	129	64	168	147^***^	179	108^***^	89	56^***^
2009	129	38	165	118^***^	179	121^***^	82	42^***^
2010	129	57	175	141^***^	142	89^***^	147	96^***^
2011	129	42	175	140^***^	151	78^***^	60	1^***^
2012	129	41	179	144^***^	72	19^***^	90	57^***^
2013	129	54	15	0^***^	169	152^***^	89	49^***^
2014	129	37	177	148^***^	175	97^***^	56	6^***^
2015	129	37	46	5^***^	77	9^***^	61	33^***^
2016	129	35	178	144^***^	179	118^***^	76	12^***^
2017	129	32	180	159^***^	60.5	27^***^	29	1^***^
2018	129	45	37	7^***^	143	123^***^	92	14^***^

**Notes.**

* P-value < 0.5.

** P-value < 0.1.

*** P-value < 0.001.

**Figure 4:**
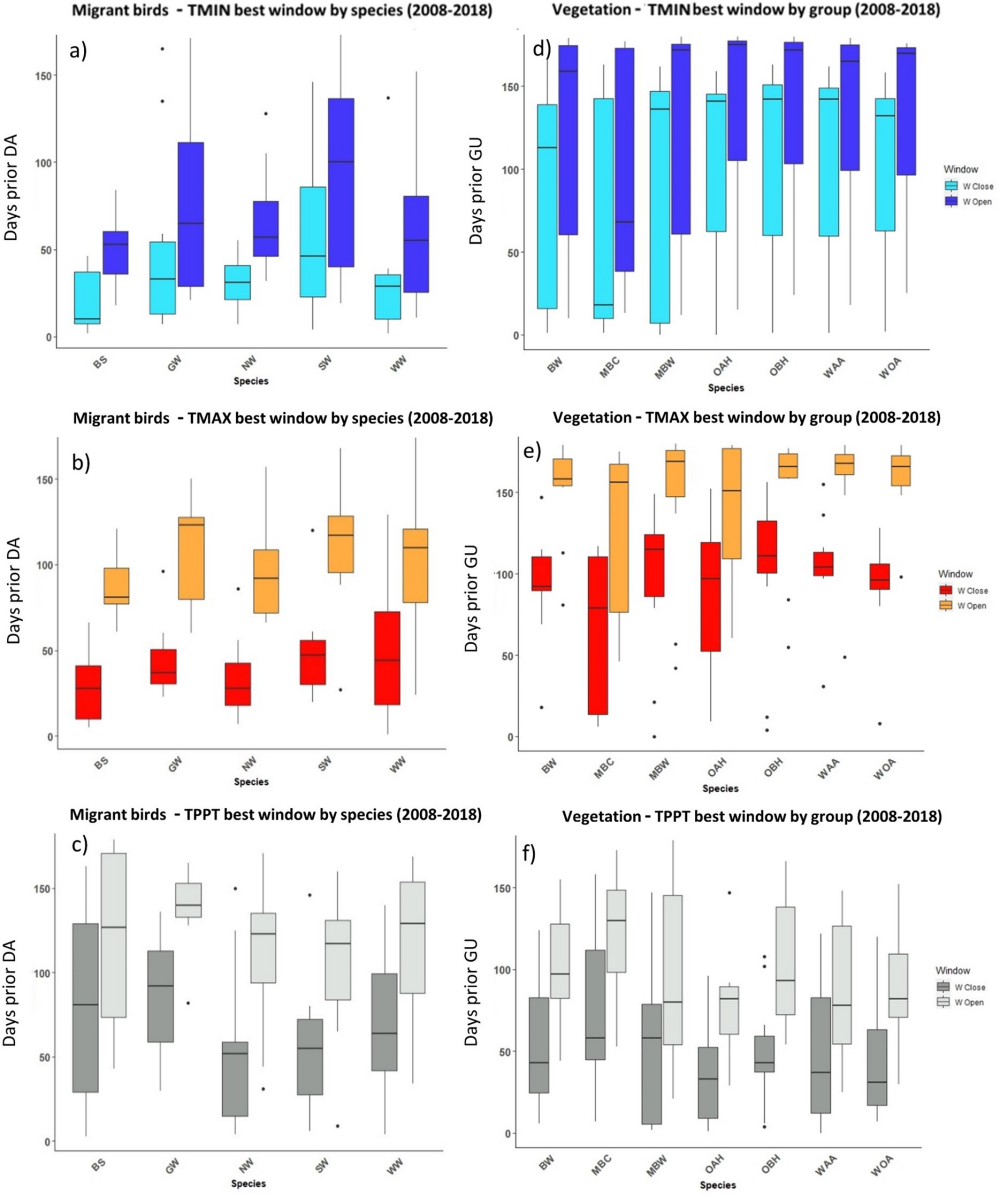
The most influential critical time windows derived from the SWR models performed with climwin for migrant birds (A–C) and vegetation groups (D–F) over the period 2008–2018. Time windows are shown by meteorological driver, TMIN (A, D), TMAX (B, E) and TPPT (C, F), window open and close are shown on different colours as indicated in their respective legends. X axis shows species code, Y axis shows the number of days prior to DA and GU, in which window open or close took place. Boxes represent the range of dates expressed in Julian days (ordinal days), whiskers represent the maximum and minimum range of values. Median values for all species window open and close are represented as horizontal black lines inside each box.

**Figure 5:**
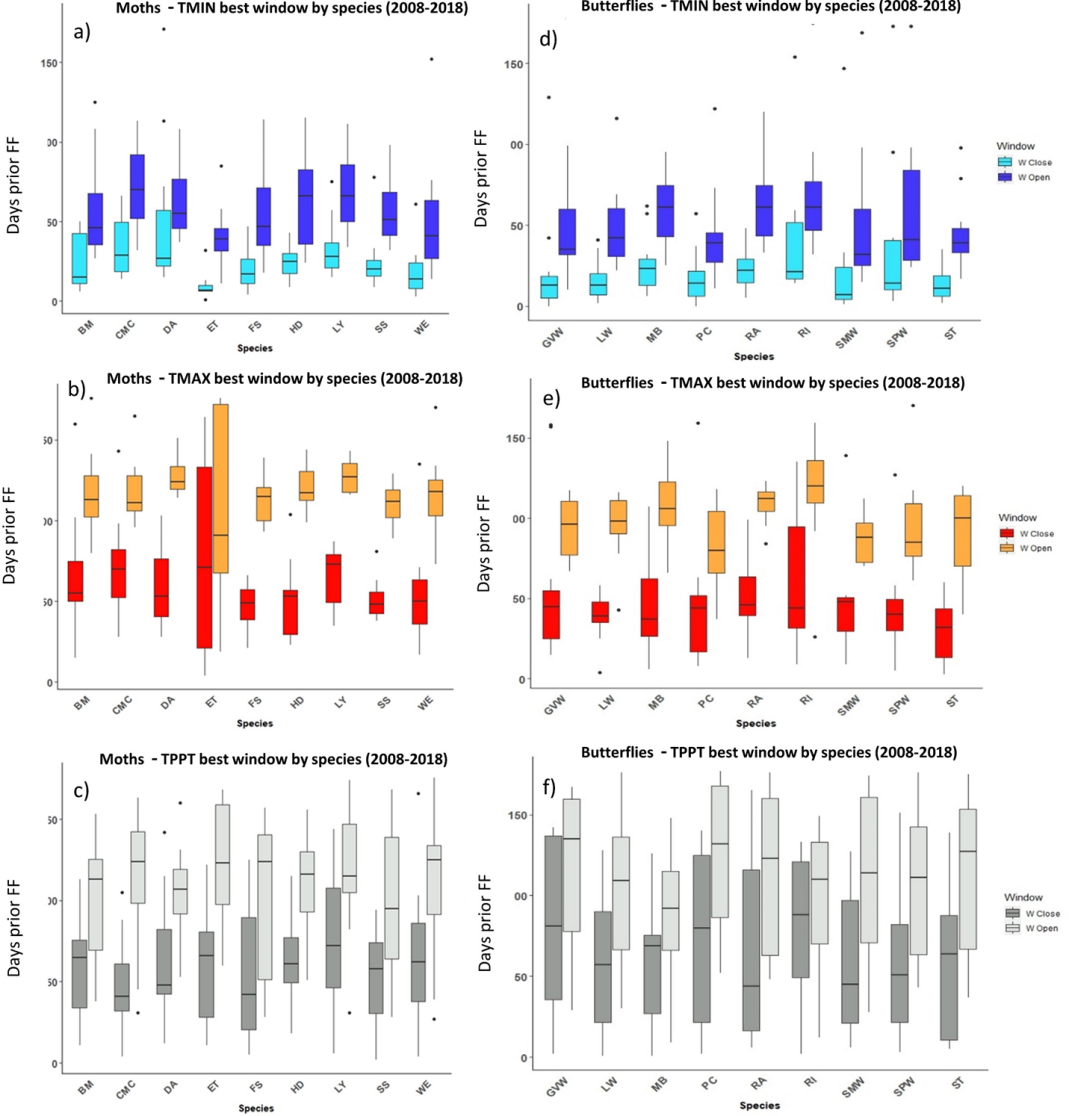
The most influential critical time windows derived from the SWR models performed with climwin for moths (A–C) and butterflies (D–F) over the period 2008–2018. Time windows are shown by meteorological driver, TMIN (A, D), TMAX (B, E) and TPPT (C, F), window open and close are shown on different colours as indicated in their respective legends. X axis shows species code, Y axis shows the number of days prior to FF in which window open or close took place. Boxes represent the range of dates expressed in Julian days (ordinal days), whiskers represent the maximum and minimum range of values. Median values for all species window open and close are represented as horizontal black lines inside each box.

In the case of the time windows of butterflies and moths, TMIN and TMAX were often the meteorological variables that showed higher influence on first flight, while TPPT time windows had less estimated influence, sometimes no better than random (see Zenodo). Coefficients for all three meteorological variables fit for butterfly and moth phenology showed high variation among species and years, with both significant positive and negative effects on first flight (Zenodo). Time windows that influence bird date of arrival and model significance was highly variable (Zenodo), with no clear trends in model coefficients for all time windows across species (Zenodo).

The period that the meteorological time windows were open fluctuated across species and groups between 2008–2018 (Figs. 4 and 5). Woodland vegetation time windows presented the longest time lapse of critical influence of each meteorological driver across all four groups, with the longest windows corresponding with TMIN, TPPT and TMAX, respectively (Figs. 5D–5F). First flight of butterflies and moths showed a similar pattern in the way each meteorological driver influenced their time windows (Figs. 4A–4F). TMIN had a greater influence around 0-70 days prior to first flight, with the median window open (∼50 days prior) and window close (∼25 days prior) being particularly low (Figs. 4A, 4D). Among migrant bird species, the length of time windows varied (Fig. 5), with TMIN median values for most species (Fig. 5A) ranging from 55 to 25 days prior to date of arrival. For a detailed description of the time window results broken down into species across the 10-year period, see Zenodo.

#### IICWM results

We documented only 17 fully synchronous relationships across 90 combinations of birds-insects when compared to the reported vegetation synchrony (Table 3). Among these, sedge warbler and greater whitethroat were the only bird species that showed synchrony with their two immediate lower trophic levels in more than one occasion, while willow warbler showed synchrony with two species from its immediate lower trophic level and barn swallow with just one species from its immediate lower trophic level. In turn, willow warbler and barn swallow showed partial synchrony (bird-insect-producers) in one case each.

**Table 3:** IICWM index for 90 bird-insect species combinations. Qualitative AL’(x,y) values are provided to give a synchrony-asynchrony insight from a tropic level perspective, bird –insect -vegetation. AL’3,2 shows the qualitative value of synchrony (S) or asynchrony (A) of the AL’(_3,2_) index for the given combination of species. AL’2,1 shows the qualitative value of synchrony (S) or Asynchrony between the given insect species and all vegetation groups, numbers indicate total number of times insect species i was synchronous or asynchronous with all vegetation groups (i.e., AL’2,1 BS- ET = S4 A3, meaning ET was synchronous with 4 vegetation groups and asynchronous with 3 vegetation groups). IICWM indicate the positive (+), asterisk (*) and negative (-) cases.

**Species**	**AL’ 3,2**	**AL’2,1**	**IICWM**	**Species**	**AL’ 3,2**	**AL’2,1**	**IICWM**	**Species**	**AL’ 3,2**	**AL’2,1**	**IICWM**	**Species**	**AL’ 3,2**	**AL’2,1**	**IICWM**	**Species**	**AL’ 3,2**	**AL’2,1**	**IICWM**
BS - BM	A	S7	*	GW - BM	A	S7	-	NW - BM	A	S7	*	SW - BM	A	S7	*	WW - BM	A	S7	-
BS - CMC	S	A7	-	GW - CMC	S	A7	-	NW - CMC	S	A7	+	SW - CMC	S	A	-	WW - CMC	S	A7	-
BS - DA	A	S7	*	GW - DA	A	S7	-	NW - DA	A	S7	-	SW - DA	A	S7	-	WW - DA	A	S7	*
BS - ET	S	S4 A3	+	GW - ET	S	A7	+	NW - ET	S	S4 A3	-	SW - ET	S	S4 A3	-	WW - ET	S	S4 A3	+
BS - FS	A	S7	*	GW - FS	A	S7	*	NW - FS	A	S7	-	SW - FS	A	S7	*	WW - FS	A	S7	-
BS -HD	A	S7	*	GW -HD	A	S7	-	NW -HD	A	S7	-	SW -HD	A	S7	*	WW -HD	A	S7	*
BS -LY	A	S7	*	GW -LY	A	S7	*	NW -LY	A	S7	*	SW -LY	A	S7	*	WW -LY	A	S7	-
BS - SS	A	S7	-	GW - SS	A	S7	*	NW - SS	A	S7	*	SW - SS	A	S7	-	WW - SS	A	S7	*
BS - WE	A	S7	*	GW - WE	A	S7	*	NW - WE	A	S7	*	SW - WE	A	S7	-	WW - WE	A	S7	*
BS -GVW	A	S7	-	GW -GVW	S	S7	+	NW -GVW	A	S7	-	SW -GVW	S	S7	-	WW -GVW	A	S7	-
BS-LW	A	S7	-	GW-LW	S	S7	-	NW-LW	A	S7	*	SW-LW	A	S7	+	WW-LW	A	S7	*
BS-MB	A	S7	-	GW-MB	A	S7	-	NW-MB	A	S7	*	SW-MB	A	S7	-	WW-MB	A	S7	-
BS –PC	A	S7	*	GW - PC	S	S7	+	NW - PC	A	S7	-	SW - PC	S	S7	+	WW - PC	A	S7	*
BS –RA	A	S7	*	GW - RA	S	S7	+	NW - RA	A	S7	*	SW - RA	S	S7	-	WW - RA	A	S7	*
BS –RI	A	S7	*	GW - RI	A	S7	-	NW - RI	A	S7	*	SW - RI	A	S7	-	WW - RI	A	S7	-
BS –ST	A	S7	-	GW - ST	S	S7	-	NW - ST	A	S7	*	SW - ST	S	S7	+	WW - ST	A	S7	-
BS - SMW	A	S7	-	GW - SMW	S	S7	+	NW - SMW	A	S7	*	SW - SMW	S	S7	-	WW - SMW	A	S7	-
BS - SPW	A	S7	*	GW - SPW	S	S7	-	NW - SPW	A	S7	*	SW - SPW	S	S7	+	WW - SPW	A	S7	**-**

Twelve instances of IICWM combinations were positive (+), which represents a bird-insect combination that is synchronous, and situations where bird time windows take place further in the past and/or insect windows take place closer to first flight. Most of these positive instances matched with synchronous combinations between birds, insects, and vegetation, while three of these combinations showed asynchrony between two immediate levels (mainly insect-vegetation) and two showed partial asynchrony (S4A3).

Most (42 out of 90) of the IICWM combinations reported a negative scenario (-), from which only five cases presented total synchrony between the three trophic levels, but not with the meteorological windows, while the remaining cases showed asynchrony between two trophic levels, mainly bird-insect combinations. Finally, we documented 36 (*) cases which represented a bird-insect asynchronous combination together with bird time windows taking place closer to date of arrival or insect time windows taking place further back before first flight. In our results, all asynchronies were between the upper levels (bird-insect) while the lower ones were synchronic in all cases (insect-vegetation).

## Discussion

The main aim of this research was to investigate spatiotemporal asynchrony/synchrony between trophic levels while determining the importance of meteorological drivers in phenological co-existence. In doing so, we aimed to address recent calls in the literature to support decision-making related to the management and conservation of species interactions, particularly related to shifting phenologies under climate change (de la Torre Cerro & Holloway, 2021; Inouye, 2022; Bailey et al., 2022; Harvey et al., 2023). This is, to our knowledge, the first study to investigate the effect of multiple trophic levels and meteorological drivers at a daily scale on the phenology of many connected species.

Our results showed a tendency towards synchrony among the study species (Table 3, Fig. 2); however, this was asymmetric within trophic levels (Figs. 2–3). Birds and insects were mainly synchronous with vegetation, with only two insects (common marbled carpet and early thorn) showing asynchrony with vegetation green-up. In multi-canopy woodlands, such as those analysed here, mixed signals early in the season can reflect predominantly understorey development before the canopy closes (Doktor et al., 2008; Helman, 2018). It is therefore not possible to relate phenology of individual plant species to animal phenology. Instead, this study considers vegetation green-up overall as a primary source of food for the animal consumers.

In contrast to synchrony between most of the animal species with vegetation, AL’_(3,2)_ showed a higher degree of asynchrony between primary- and secondary-consumers (Fig. 3). We identified a trend towards phenological asynchrony within migrants and their prey in agreement with previous research (Both et al., 2010; Mallord et al., 2017; Mayor et al., 2017); however, the degree of asynchrony varied among bird-insect combinations and, overall, only a few combinations were highly asynchronous (Fig. 3). The relevance of asynchrony among species found in our results must be taken with caution as (1) not all species are interdependent and (2) species-specific traits can buffer negative impacts. Such buffering could occur if a bird species’ dietary range is wide enough to allow it to switch to other prey (Donnelly, Geyer & Yu, 2015; Mallord et al., 2017). Despite this, our results potentially point to a concerning trend of asynchrony, which was observed across most combinations, suggesting future research is warranted to quantify the extent of this potential trophic cascade.

Our results also suggested that date of arrival of migrant birds followed green-up in all cases (Table 3, Fig. 2), corroborating the findings of Mayor et al. (2017). However, our results suggest that the birds might not be tracking green-up events at a sufficient pace. This was supported by cases where migrant birds and vegetation were classed synchronous by our AL’ index, but the high values (close to 1) indicated a big gap between green-up and date of arrival. In some cases, this could reflect delayed synchrony, leading to possible asynchronies. For Lepidoptera, most species showed AL’ synchrony values with green-up, but again some species were more synchronous than others. This might be explained by species specific life history-traits, such as overwintering strategies that influence the time gap between green-up and first flight. For example, ringlet, dark arches, and common marbled carpet overwinter as larvae, presenting high gaps between adult first flight and green-up, while early thorn overwinters as pupa and its first flight showed high degree of synchrony with green-up.

These asynchronies can have significant impacts on the trophic network, including trophic cascades. With migrant birds arriving earlier on average each year (sometimes as early as March; Zenodo), they are arriving oftentimes before the first flight of their insect prey. This can result in a lack of food for these birds, impacting their fitness and capacity to breed (Reed, Jenouvrier & Visser, 2013). Such shifts in the phenological network highlight the importance of enacting conservation efforts that can widen the inter-dependency among species, especially in the case of asynchrony. For example, the targeted promotion of earlier, later, or extended initiatives such as ‘No Mow May’ to coincide with maximal nectar sugar resources for pollinators (Hemmings, Elton & Grange, 2022), and subsequently maximal pollinator numbers (*i.e.,* more insects), alongside projected arrival dates of migrant birds could buffer the impact of any trophic mismatch.

The addition of the meteorological factors in our IICWM corroborated the importance of such variables giving context to synchrony-asynchrony indices to fully understand how species are interacting in relation with changes in the environment. Our IICWM index showed high variation in the time windows that birds and insects followed over the study period (Table 3). There was high inter-annual variation at group and species level, emphasising the importance to consider a dynamic conceptualisation of meteorological drivers. We found that time windows varied across groups of species and at species level, supporting the hypothesis that phenological sensitivity to meteorological variables is species-specific, and studies should thus aim to explore the impact of climate change on species accordingly.

The IICWM is predicated on the assumption that if species follow the same meteorological cues across years, values for the time window should remain relatively constant, and therefore IICWM values would be similar to AL values. However, meteorological conditions fluctuate abruptly among years (*i.e.,* extreme meteorological events), and thus we expected time windows to show this variation. Our results confirmed this, with IICWM showing high variation when compared against AL (Tables 2 and 3), indicating changes among the temporal influence of environmental drivers. Almost half of the relationships identified in the IICWM reported a negative value, which identified at least one asynchronous interaction among either birds-insects or insects-vegetation. The addition of meteorological information to interaction indices provided the mechanism to investigate the interlinked relationship between abiotic and biotic factors within phenology. Subsequently, enhanced indices like the IICWM can improve our predictive ability of trophic mismatches that could lead to biodiversity loss.

For example, colder temperatures during winter and spring have typically been correlated with a later first flight (Gezon et al., 2018), while a trend of earlier first flight has been associated for butterflies and moths with increasing temperatures worldwide (Bell et al., 2019; Cohen, Lajeunesse & Rohr, 2018; Maurer et al., 2018; van der Kolk, WallisDeVries & Van Vliet, 2016). However, inter-specific variation in sensitivity to changes in environmental conditions has been previously reported (Gezon et al., 2018; Maurer et al., 2018). This aligns with our results showing different estimated sensitivity to the three meteorological variables across species and years (Zenodo). Since small ectotherms have been demonstrated to track temperatures better than large taxa (Cohen, Lajeunesse & Rohr, 2018), we suggest that the variation in these species’ weather time windows and the peaks of early and delayed phenology found by our models may correspond with abrupt changes in temperature or with extreme meteorological events. In a recent study using daily maximum and minimum temperature and precipitation, Long et al. (2017) demonstrated that extreme climate events played a significant role in butterfly phenology, highly associated to species-specific life-history functional traits, such as overwintering stage or voltinism. Moreover, optimal synchrony windows may change with temperature increases, with new interactions created as part of these changing phenologies (Johnson et al., 2010), meaning while trophic mismatches are possible, new interactions may buffer or strengthen some interactions. Thus, extreme climate events and life-history functional traits might explain species-specific response to our meteorological drivers. Therefore, inter-annual variability in window open, close and window length might be driven by these factors.

The selection of fine-scale temporal (*i.e.,* daily, weekly) meteorological variables in phenological studies is increasingly being utilised, as it more precisely captures the variation in phenology that is explained by the relevant drivers at the appropriate temporal and spatial scale (Van De Pol et al., 2016; Holloway, Kudenko & Bell, 2018; Simmonds, Cole & Sheldon, 2019; Dai et al., 2023). We opted to use the relative sliding time window approach, as opposed to other widely used methods (*e.g.*, absolute sliding time windows) to ensure that temporal dynamism was incorporated. This temporal dynamism can reflect variation within the annual calendar of when phenological events occur, but also spatial variation in the timing of events, as species that occupy large geographic ranges often cover diverse environmental gradients that can reflect flexibility and intraspecific differences in phenology (Gallinat et al., 2021). However, relative sliding time window analysis can report highly variable results, especially when contrasted to other cue identification techniques that use absolute windows (Simmonds, Cole & Sheldon, 2019). This can result from a statistical artefact of the method, creating unexpected species-environment relationships. For example, in a model that assesses meteorological conditions 180 days prior to the phenological event, there will be more colder days in the model that occurred 180 days before March 1 than 180 days before May 1. This means the statistical approach can identify specific weather conditions that are not necessarily biologically relevant, but statistically significant (Simmonds, Cole & Sheldon, 2019).

To explore whether the variation over the individual years within the 11-year time period was congruent with the signal across the dataset compiled of all 11-years, we ran the SWR analysis for several species with aggregated data across this time period (Zenodo). Our results were constant in terms of the time windows reported with the lowest delta AICc values (Zenodo), with the exception that for butterflies, moths and birds the TMIN mean windows advanced closer to the event. Therefore, we are confident in the ability of the SWR analysis to capture the broad trends over the period. Despite this, we acknowledge that analysing annual phenological cues across time windows produces so many results that it if a specific trend is not obvious, it can be hard to interpret in a biological way (Zenodo). Subsequently, we refrain from any ecological interpretation related to shifting phenological cues. This issue is further amplified by the fact that extreme climate events are ‘softened’ in aggregated statistics but are often considered one of primary cues or drivers in ecological research (Johansson et al., 2020). Time-series analysis that quantifies the periodic or sinusoidal variations found in the metrological data (*e.g.*, exponential moving average, frequency of max peak) could resolve the methodological challenge associated with the subjective decision of defining time window length. This could mean that extreme events or cues are accounted for in statistical parameterization. Despite this, the relative sliding time window approach provided us with the opportunity to incorporate a dynamic representation of meteorology in the IICWM, which was the primary aim of this research, supporting a novel application of biodiversity conservation.

Due to the spatiotemporal nature of our study, we opted to use the first recorded value of phenology, such as date of arrival and first flight. We explored the use of percentiles (*i.e.,* 5th, 10th first flight date) instead of the earliest value as has been used in phenological research (Bell et al., 2015); however, due to the uneven samples within each 10 km grid, and the fact that many grids had less than 10 observations, this meant all percentile values under the 10th would have been the same. Unbalanced data collated as part of citizen science projects are well recognised, along with their potential to support national climate and ecosystem assessments, including phenology (Crimmins & Crimmins, 2022). While accounting for spatial bias in citizen science data is well established (Millar, Hazell & Melles, 2019), temporal bias is perhaps more limited and is often thought of analogous to spatial bias (Callaghan et al., 2019). Temporal biases across seasons, particularly for phenology data are obvious (Arab, Courter & Zelt, 2016), but even biases towards weekend observations have been noted (Courter et al., 2013). Therefore, future research needs to identify methods of bridging the spatial and temporal gaps in citizen science data, such that larger datasets of phenology (or any ecological phenomenon) can be utilised to account for ecological uncertainty.

The effect of weather conditions at breeding grounds influencing date of arrival has been argued to be of little relevance particularly for long-distance migrants, while short-distance migrants or species that migrate at a slower pace have been suggested to better track environmental conditions at destination habitats (Hurlbert & Liang, 2012; Chmura et al., 2019; Kullberg et al., 2015). However, some studies argue that long-distance migrants are also capable of keeping pace with changing climatic conditions, advancing their arrival time to breeding grounds because of phenological mismatch with food sources at these breeding grounds, shorter stopovers or through mechanisms such as micro-evolution or photoperiodic cues (Chmura et al., 2019; Helm et al., 2019; Jonzén et al., 2006; Jonzén, Hedenström & Lundberg, 2007; Kolářová et al., 2017). Therefore, the choice of migrant birds and the phenological indicator of date of arrival may not necessarily reflect the most relevant aspect of phenology in terms of trophic interactions. For example, higher abundances of insects may be more important during periods of chick raising rather than upon arrival (Emmenegger, Hahn & Bauer, 2014), with studies noting that seabird egg-laying and hatching does not correspond to temperature changes (Merkel et al., 2019). Date of arrival of migrant birds can be considered a proxy, and we refrain from commenting on the role of meteorological variables in determining these dates to prevent compounding understanding of their ecology. However, due to the sparsity on national scale datasets, regarding other life-history traits, such as egg-laying dates or hatching dates, particularly in Ireland we opted to incorporate these into our models. This supported the development of a novel multi-level trophic interaction index, that could be replicated in different systems across any phenological indicator. Therefore, the widely applied assumption that it is meteorological conditions alone that are driving phenology should be revisited, with research needed to incorporate and disentangle the role of both abiotic and biotic drivers in phenology.

## Conclusions

Phenological mismatches between interlinked species can have negative impacts for biodiversity, ecosystems, and the trophic network. Here we developed novel interaction indices that quantified the level of synchrony and asynchrony among groups of species in three interlinked trophic levels, as well as accounting for a dynamic representation of meteorology. The use of the relative sliding time window approach identified the critical time windows of meteorology that influenced phenology, highlighting the potential to incorporate both abiotic and biotic factors in such indices. The new indices of phenological change identified several asynchronies within trophic levels, allowing exploration of potential interactions based on synchrony among interlinked species. While most species combinations were synchronous as per the results shown by our synchrony-asynchrony indices, we found asynchronies typically between migrant birds and insects, and a possible effect of “delayed synchrony” in some bird-woodland vegetation combinations. Our novel index of synchrony-asynchrony including a meteorological dimension could be highly informative and should open new pathways for studying synchrony among species and interaction networks.

## Additional Information and Declarations

### Data Availability

The following information was supplied regarding data availability: The code, an example of the processed data for a moth species in 2008, and a shapefile of the fishnet grid are available at Zenodo: Holloway, P. (2024). Processed Data, Code, & Supplementary Material for de la Torre Cerro et al. (2024) Modelling asynchrony in phenology using a dynamic representation of meteorological variables [Data set]. Zenodo. https://doi.org/10.5281/zenodo.13991620. The bird data is available at ebird: https://ebird.org/science/use-ebird-data/download-ebird-data-products. The BirdTrack data is available at: https://www.bto.org/our-science/projects/birdtrack. The moth data is available at MothsIreland: http://www.mothsireland.com/maps-overview/maps-macro-english. The butterfly is available at the National Butterfly Monitoring Scheme: https://maps.biodiversityireland.ie/Dataset/316. The vegetation data is available at Landsat: https://espa.cr.usgs.gov. The meteorological data is available at Met Éireann: https://www.met.ie/climate/available-data/mera.
